# Combined epigenetic/genetic study identified an ALS age of onset modifier

**DOI:** 10.1186/s40478-021-01183-w

**Published:** 2021-04-23

**Authors:** Ming Zhang, Zhengrui Xi, Sara Saez-Atienzar, Ruth Chia, Danielle Moreno, Christine Sato, Mahdi Montazer Haghighi, Bryan J. Traynor, Lorne Zinman, Ekaterina Rogaeva

**Affiliations:** 1 Shanghai First Rehabilitation Hospital, School of Medicine, Tongji University, Shanghai, 200090 China; 2 Tanz Centre for Research in Neurodegenerative Diseases, University of Toronto, 60 Leonard Ave., Toronto, ON M5T 0S8 Canada; 3 Clinical Center for Brain and Spinal Cord Research, Tongji University, Shanghai, 200092 China; 4 Institute for Advanced Study, Tongji University, Shanghai, China; 5 Neuromuscular Diseases Research Section, Laboratory of Neurogenetics, National Institute on Aging, National Institutes of Health, Bethesda, MD 20892 USA; 6 Sunnybrook Health Sciences Centre, 2075 Bayview Ave, Toronto, ON M4N 3M5 Canada; 7 Division of Neurology, Department of Medicine, University of Toronto, Toronto, Canada

**Keywords:** ALS, Age of onset, Modifier, DNA methylation, CpG-SNPs, Genetic association

## Abstract

Age at onset of amyotrophic lateral sclerosis (ALS) is highly variable (eg, 27–74 years in carriers of the G_4_C_2_-expansion in *C9orf72*). It might be influenced by environmental and genetic factors via the modulation of DNA methylation (DNAm) at CpG-sites. Hence, we combined an epigenetic and genetic approach to test the hypothesis that some common single nucleotide polymorphisms (SNPs) at CpG-sites (CpG-SNPs) could modify ALS age of onset. Our genome-wide DNAm analysis suggested three CpG-SNPs whose DNAm levels are significantly associated with age of onset in 249 ALS patients (q < 0.05). Next, genetic analysis validated the association of rs4970944 with age of onset in the discovery (n = 469; P = 0.025) and replication (n = 4160; P = 0.007) ALS cohorts. A meta-analysis of the cohorts combined showed that the median onset in AA-carriers is two years later than in GG-carriers (n = 4629; P = 0.0012). A similar association was observed with its tagging SNPs, implicating a 16 Kb region at the 1q21.3 locus as a modifier of ALS age of onset. Notably, rs4970944 genotypes are also associated with age of onset in *C9orf72*-carriers (n = 333; P = 0.025), suggesting that each A-allele delays onset by 1.6 years. Analysis of Genotype-Tissue Expression data revealed that the protective A-allele is linked with the reduced expression of *CTSS* in cerebellum (P = 0.00018), which is a critical brain region in the distributed neural circuits subserving motor control. *CTSS* encodes cathepsin S protein playing a key role in antigen presentation. In conclusion, we identified a 16 Kb locus tagged by rs4970944 as a modifier of ALS age of onset. Our findings support the role of antigen presenting processes in modulating age of onset of ALS and suggest potential drug targets (eg, *CTSS*). Future replication studies are encouraged to validate the link between the locus tagged by rs4970944 and age of onset in independent ALS cohorts, including different ethnic groups.

**Supplementary Information:**

The online version contains supplementary material available at 10.1186/s40478-021-01183-w.

## Introduction

Amyotrophic lateral sclerosis (ALS) is characterized by the progressive degeneration of upper and lower motor neurons in the brain and spinal cord, leading to paralysis [6]. About 90% of patients have sporadic ALS. Genetic mutations explain 10–20% sporadic and ~ 50% familial ALS, mainly caused by the most common ALS genes (*C9orf72, SOD1, TARDBP* and *FUS*) [6, 30]. Patients with ALS have variable clinical presentation, including disease duration and age or site of onset [30]. For example, carriers of the G_4_C_2_-expansion in *C9orf72* have been reported to have disease onset between 27 and 74 years and duration of 0.5–22 years [10].

Disease phenotype can be modified by DNA methylation (DNAm) at CpG dinucleotides, which is one of the key epigenetic modifications regulating gene expression or RNA splicing. For instance, smoking and head injury (suggested ALS risk factors [8, 31]) are linked to DNAm [20]. Furthermore, DNAm is closely associated with aging-the strongest risk factor of ALS [3]. Specifically, the cumulative assessment of DNAm levels at 353 age-related CpGs (constituting DNAm-age) revealed an association of DNAm-age acceleration with disease age of onset, duration or survival in *C9orf72*-carriers and general ALS patients [35, 36]. DNAm-age is not greatly modulated by genetic variations, because none of the 353 age-related CpGs are mapped to common single nucleotide polymorphisms (SNPs) with a minor allele frequency (MAF) > 5% [3].

In general, DNAm levels at certain GpGs could be modified by genetic factors. The strong genetic control of DNAm is evident by the very similar methylome pattern in identical twins/triplets vs fraternal siblings [33, 37]. A specific example is increased DNAm at the *C9orf72* locus in response to a G_4_C_2_-expansion, which correlates with disease duration and age of onset [14, 27, 32]. However, it is largely unknown if other genetic variants that alter DNAm are linked to ALS phenotypes.

Importantly, CpG-sites are mutational hotspots, because methyl-C can spontaneously deaminate to T [19]. SNPs causing the gain/loss of CpG-sites (CpG-SNPs) are linked to DNAm level and could modify disease phenotype [34]. CpG-SNPs contribute largely to allele-specific methylation, which is linked to gene expression, transcription factor binding, and associated with some mental illnesses (eg, schizophrenia) [13, 15]. Our prior study of CpG-SNPs revealed the *C6orf10* locus as an age of onset modifier in *C9orf72*-carriers, but not in *C9orf72* negative ALS patients [34]. SNPs in *C6orf10* are linked with the frontal cortex expression of *HLA-DRB1* (a proinflammation and antigen-presenting gene) [34]. It is unknown if expression quantitative trait loci (eQTL) of other antigen-presenting genes are linked with ALS age of onset, which was in part addressed in the current study.

Here, we used an integrated epigenetic and genetic approach to identify functional genetic variants associated with age of onset in ALS patients.

## Materials and methods

### ALS participants

Cohort characteristics of unrelated ALS patients are presented in Table 1. The discovery cohort included 469 Canadian ALS patients (without causal mutations in *C9orf72, SOD1, TARDBP* or *FUS*). The replication cohort consisted of 4160 US ALS patients, including 333 *C9orf72*-carriers. All patients are of Caucasian origin and diagnosed with ALS using the El Escorial revisited clinical criteria [5]. ALS age of onset was defined as the self-reported age at which the first limb (spinal) or bulbar symptom appeared.

**Table 1:** Sample characteristics for unrelated ALS patients included in the DNA methylation (DNAm) study, and genotyping analysis of candidate variants in discovery and replication stage

Sample characteristics	DNAm analysis (n = 249)	Genotyping analysis (n = 469)	Replication cohort (n = 4160)
Familial ALS (n, %)	49, 19.6%	68, 14.5%	415, 10.0%
Sporadic ALS (n, %)	200, 80.3%	401, 85.5%	3745, 90%
Sex, male (n, %)	148, 59.4%	280, 59.7%	2517, 60.5%
Median age of onset (interquartile range), years	60 (51–69)	61 (52–68)	58 (49–66)
Bulbar site of onset (n)	67	112	990
Limb site of onset (n)	172	314	2882

### Procedures

We analyzed genome-wide DNAm data of ~ 850,000 DNAm-sites from the EPIC BeadChip (Illumina) previously generated using bisulfite converted blood DNA of Canadian ALS patients [35]. We used the minfi package in R-project [2] to pre-process the raw data and select common CpG-SNPs (MAF > 5%). The β-value was used to estimate the DNAm level of each CpG-site. We included common CpG-SNPs with a difference between maximum and minimum β-value > 0.5 (considering the effect of SNPs on DNAm) and used the gaphunter function (minfi package) [1] to study CpGs with a multimodal distribution of DNAm level.

For the genetic analysis, we used blood DNA. In the discovery cohort, genotypes for candidate SNPs were obtained by multiplex genotyping using iPlex (Agena Bioscience) and MassArray Analyzer 4 at the Clinical Genomics Centre (Toronto, Canada). For the replication cohort, genome-wide genotyping was performed in the Laboratory of Neurogenetics, National Institutes of Health using HumanOmniExpress (version 1.0 genotyping 716,503 SNPs) according to the manufacturer's protocol (Illumina Inc., San Diego, CA). The 34,335 US controls (71% females; mean age 65 with a standard deviation of 13 years) were previously genotyped on HumanOmni BeadChips (Illumina) [23]. In the replication stage, 4 SNPs (Table 2) were either genotyped or imputed using the Michigan Imputation Server pipeline employing Minimac4 [12] based on the Haplotype Reference Consortium r1.1 2016 [22] (http://www.haplotype-reference-consortium.org).

**Table 2:** The association of rs4970944 and its tagging variants with ALS age of onset in the discovery and replication cohorts

SNP	Location (hg19)	MAF	Discovery stage (n = 469)			Replication stage (n = 4160)		
			B	SE	*P* value	B	SE	*P* value
rs4970944	chr1:151,163,317	0.32	2	0.9	0.025	0.8	0.3	0.007
rs10888406	chr1:151,166,896	0.32	2	0.9	0.025	0.8	0.3	0.007
rs11204785	chr1:151,150,857	0.33	NA	NA	NA	0.7	0.3	0.013
rs11807075	chr1:151,153,806	0.33	NA	NA	NA	0.7	0.3	0.014

Results from the additive multivariate linear regression model are presented (adjusted for sex, ALS site of onset and family history). Non-Finnish European minor allele frequencies (MAF) were extracted from the gnomAD database (NA = not available, B = linear regression coefficient Beta, SE = standard error)

To measure the degree of linkage disequilibrium (LD), we extracted R^2^ values (range from 0 to 1, indicating the highest LD) from the LDlink web tool by selecting the 1000 Genomes European population data (https://ldlink.nci.nih.gov/?tab=home). We searched for known variants within the boundaries of the LD-block (R^2^ > 0.9) tagged by the top significant SNP (rs4970944) using the ‘proxy search’. The LD-block was also analyzed for transcription factor binding sites and DNase I hypersensitivity using the UCSC genome browser. We searched for eQTL using Genotype-Tissue Expression data (GTEx v8) from 49 types of human tissue [9]. We used the GTEx portal (https://www.gtexportal.org/) to analyze the association between rs4970944 genotypes and gene expression in specific tissues by a linear regression method. Normalized effect size (NES) was defined as the slope of the linear regression.

To understand which cell types are linked to *CTSS* gene expression in brain tissue, we analyzed publicly available single nuclei RNA sequencing data of 8 human entorhinal cortex samples (GEO: GSE138852). The Seurat package [7] in R was used to perform quality control, pre-processing, normalization, and dimensional reduction/clustering by the Uniform Manifold Approximation and Projection (UMAP) technique.

### Statistics

We used the linear regression model in R to assess the genome-wide association between DNAm levels of CpG-SNPs and ALS age of onset, and calculated the false discovery rate to obtain adjusted q-values [34]. Adjustments for sex, site of onset, DNAm-age acceleration and the top 5 principal components (PCs) were also performed. The PCs were generated by PC analysis of common CpG-SNPs with the prcomp function in R. The Manhattan plot was used to prioritize significant CpG-SNPs (q < 0.05) for further genetic study. A QQ plot was generated and the genomic inflation factor was estimated, using the R qqman package [11].

To analyze the association between genotypes and age of onset, we used an additive multivariate linear regression model adjusting for sex, site of onset, family history, and/or DNAm-age acceleration. We presented the linear regression coefficient Beta (B) with standard error (SE). We used the Mann Whitney U test to evaluate differences in age of onset in the ALS subgroups (Table 1) stratified by sex (males vs females), site of onset (limb vs bulbar onset), ALS family history (familial vs sporadic) or *C9orf72* status (expansion vs wild-type). We also conducted a meta-analysis (R metafor package) [34] with a fixed-effect model to assess the pooled effect size of the coefficient B for the discovery and replication stages. R project 4.0.0 was used for statistical analysis. Results with a *P*-value < 0.05 were accepted as statistically significant.

## Results

### Discovery stage suggested candidate CpG-SNP linked to ALS age of onset

**Fig. 1:**
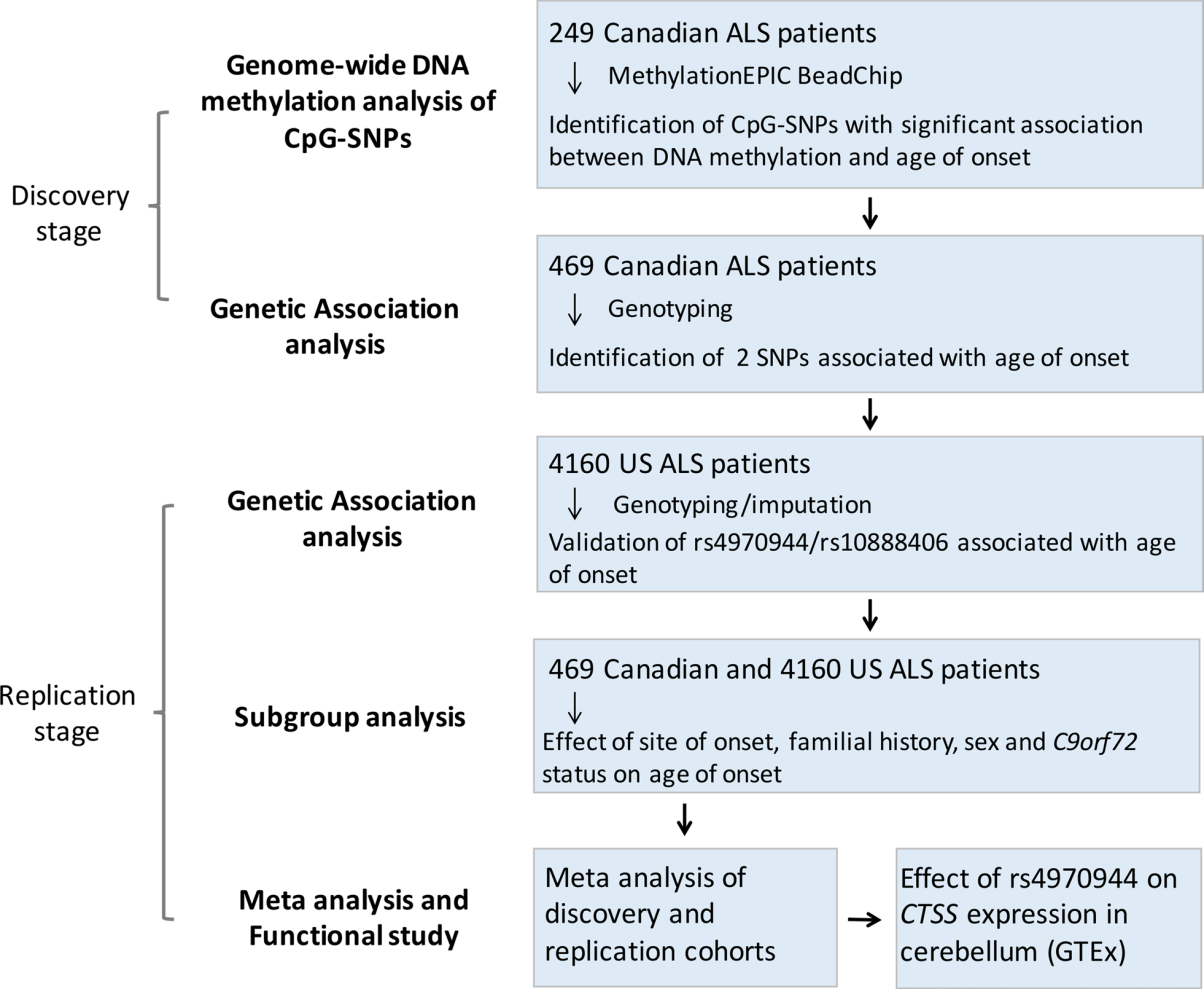
Flowchart of the study design

**Fig. 2:**
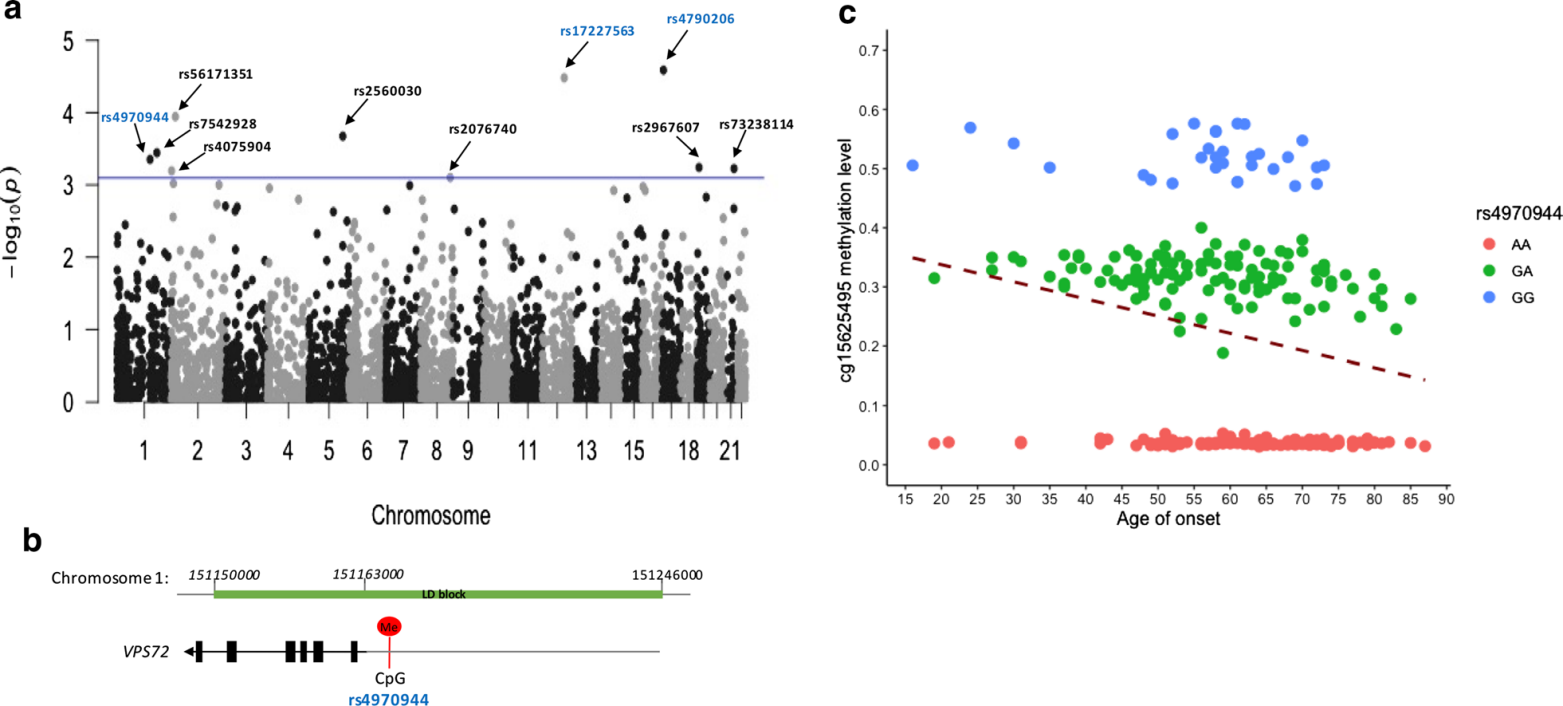
Genome-wide DNA methylation (DNAm) analysis and genetic association study suggested candidate CpG-SNPs associated with ALS age of onset.  **a** Manhattan plot presenting the association between DNAm levels of common CpG-SNPs and ALS age of onset. Three CpG-SNPs in blue remained significant after adjusting for sex, site of onset, DNAm-age acceleration and the top 5 principal components. The blue line represents the trend of statistical significance (false discovery rate < 0.05). **b** The locus on chr1:151,150,000–151,246,000 tagged by rs4970944 associated with ALS age of onset. Arrows indicate the transcriptional direction of *VPS72* (5′ to 3′). “Me” in red represents the CpG-site, methylation of which is affected by rs4970944. The linkage disequilibrium (LD) block tagged by rs4970944 (R^2^ > 0.8) is highlighted in green. **c** Genotypes of rs4970944 are significantly associated with DNAm status: P = 2 × 10^−16^, B = -0.25 (SE: 0.003); and age of onset: P = 0.003 adjusted for sex, family history, site of onset and DNAm-age acceleration, B = 3.0 (SE: 1.0). The dashed line represents the linear regression trend

The study design is presented in Fig. 1. First, we conducted a genome-wide DNAm analysis of CpG-SNPs in 249 Canadian ALS patients (Table 1). Among the 4300 common CpG-SNPs with a multimodal distribution, the DNAm levels at 10 CpG-SNPs were associated with age of onset at a false discovery rate < 0.05, three of which remained significant after adjustment for sex, site of onset, DNAm-age acceleration and the top 5 PCs (Fig. 2a, b, Additional file 1: Table S1, Fig. S1).

**Fig. 3:**
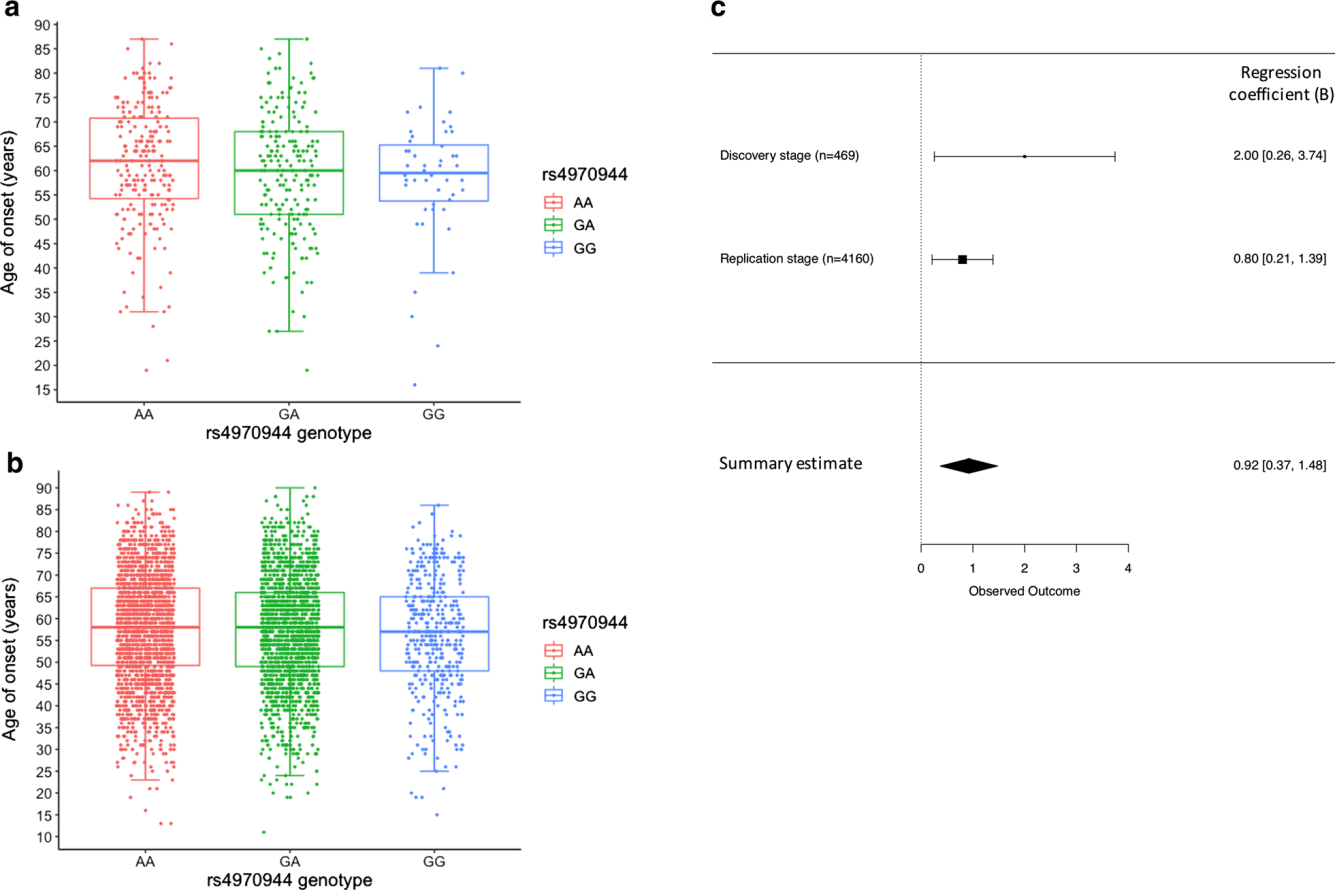
The association between rs4970944 and ALS age of onset. **a** Genotypes of rs4970944 are significantly associated with age of onset in the discovery cohort of 469 Canadian ALS patients (P = 0.025, adjusted for sex, site of onset and family history, B = 2.0, SE = 0.9). Each A-allele is associated with a 2-year later onset. **b** Genotypes of rs4970944 are significantly associated with age of onset in the replication cohort of 4160 US ALS patients (P = 0.007, adjusted for sex, site of onset and family history; B = 0.8, SE = 0.3). Each A-allele is associated with a 0.8-year later onset. **c** Meta-analysis of the adjusted regression coefficient from the discovery cohort (n = 469) and the replication cohort (n = 4160) confirmed the significant association between rs4970944 and ALS age of onset (pooled B = 0.9, P = 0.0012)

Next, we conducted a genetic analysis of candidate variants detected during the DNAm study in an expanded Canadian ALS cohort (n = 469; Table 1). Only rs4970944 and its tagging SNP (rs10888406) showed significant association with ALS age of onset (Table 2). Each A-allele of rs4970944 is linked to a 2-year later onset (adjusted P = 0.025, B = 2.0, SE = 0.9) (Fig. 3a, Table 2). In the original subgroup with DNAm data (n = 249), multivariate linear regression analysis confirmed that the genotypes of rs4970944 are associated with both DNAm level and age of onset (Fig. 2c). Specifically, rs4970944 genotypes control the gain or loss of DNAm at CpG-site cg15625495 (P = 2 × 10^−16^), thereby underlying the association with age of onset (adjusted P = 0.003, B = 3.0, SE = 1.0).

### Replication stage validated the link of ALS age of onset with LD-block tagged by rs4970944

In a US cohort of 4160 ALS patients (Table 1), we validated the association with age of onset for rs4970944 after adjustment for sex, site of onset, and family history (P = 0.007, B = 0.8, SE = 0.3), which suggested that each A-allele of rs4970944 delays ALS onset by 0.8 year (Table 2, Fig. 3b). Four SNPs in strong LD with rs4970944 (R^2^ > 0.9) implicate a 16 Kb LD-block (chr1:151,150,857–151,166,896) partially overlapping *VPS72* (Additional file 1: Fig. S2). Genotypes for three of them (rs10888406, rs11204785, rs11807075) were available in the replication dataset and associated with age of onset (adjusted P = 0.007–0.014; B = 0.7–0.8, SE = 0.3) (Table 2, Additional file 1: Fig. S3). Notably, the 16 Kb LD-block tagged by rs4970944 is associated with ALS age of onset, but not with ALS risk in our case–control study (4160 US ALS patients and 34,335 controls; P = 0.93).

### Subgroup analyses and the association of rs4970944 with ALS age of onset in *C9orf72*-carriers

We performed a subgroup analysis for sex, family history and site of onset in the discovery and replication cohorts. We observed a significantly younger onset in ALS patients with limb vs bulbar onset (P = 0.0016 for Canadian patients; P = 2.2 × 10^–16^ for US patients) or with familial vs sporadic ALS (P = 0.0005 for Canadian patients; P = 3.1 × 10^–13^ for US patients) (Additional file 1: Fig. S4-S5, Table S2). Males showed a significantly younger onset than females in the US cohort (P = 0.001, Additional file 1: Fig. S5, Table S2), but not in the more modest Canadian cohort (P = 0.7, Additional file 1: Fig. S4, Table S2). Notably, a subgroup analysis for the 4030 cases with known status for the expansion in *C9orf72* did not detect a significant difference in age of onset between *C9orf72*-carriers (n = 333) vs non-carriers (Additional file 1: Fig. S6a).

After adjustment for sex, site of onset and ALS family history, rs4970944 is significantly associated with age of onset in both *C9orf72*-carriers (P = 0.025, B = 1.6, SE = 0.7; n = 333) and non-carriers (P = 0.015; B = 0.8, SE = 0.3; n = 3697) (Additional file 1: Fig. S6b-6c). It suggested that each A-allele of rs4970944 delays ALS onset by 1.6 years in *C9orf72*-carriers. The median onset in AA-carriers is 2.5 years later than GG-carriers: 57.5 years with an interquartile range (IQR) of 52–64 vs 55.0 years (IQR: 50–59). The genotypes of tagging SNPs (rs10888406, rs11807075, rs11204785) showed a similar association with ALS onset in *C9orf72*-carriers (P = 0.018–0.025, B = 1.6–1.7, SE = 0.7).

### Meta/pooled-analyses revealed overall effect of rs4970944 tagging SNPs on ALS age of onset

To estimate the overall effect size of rs4970944 on age of onset, we conducted a meta-analysis of the adjusted coefficient Beta (B) in the 4629 ALS patients from the discovery and replication stages using a fixed-effects model. We observed that every A-allele of rs4970944 is linked to 0.9-year later onset (pooled B = 0.9, P = 0.0012) (Fig. 3c), and the same result was obtained in the 4166 *C9orf72* negative patients (pooled B = 0.9, P = 0.0015, Additional file 1: Fig. S8). A pooled linear regression analysis suggested a similar effect of the A-allele on age of onset (adjusted P = 0.001, B = 0.9, SE = 0.3) (Additional file 1: Fig. S7). The median onset in AA-carriers was 2 years later than GG-carriers: 59 years (IQR:50–76) vs 57 years (IQR:49–65). The same result was observed for tagging SNP rs10888406 (Additional file 1: Fig. S3).

### Genotypes of rs4970944 are associated with microglia related *CTSS* expression in cerebellum

We conducted a bioinformatics analysis of the top SNPs tagged by rs4970944 (R^2^ > 0.9) and found that none of them overlap transcription factor binding sites or DNase I hypersensitivity sites (Additional file 1: Table S3). To explore if rs4970944 genotypes could modify ALS age of onset by regulating gene expression, we analyzed the GTEx eQTL dataset [9], consisting of 49 tissues from up to 670 individuals. We did not observe an association of rs4970944 with expression of *VPS72* (the gene partially overlapping the LD-block tagged by rs4970944), which is not surprising since the closest gene to the significant variant is often not the functional disease-gene [18, 34]. However, rs4970944 genotypes are associated with the expression of adjacent genes in a wide range of tissues (Additional file 1: Table S4). Importantly, the A-allele of rs4970944 (linked to a later ALS onset) is significantly associated with reduced expression of *CTSS* in cerebellum (P = 0.00018, NES = −0.31, n = 209; Additional file 1: Fig. S9), with similar results for its tagging SNPs (P = 0.00018, NES = −0.31 for rs10888406; P = 0.000056, NES = −0.34 for rs11204785; P = 0.000059, NES = −0.33 for rs11807075). For other ALS-related tissues available in the GTEx dataset (eg, spinal cord), we did not observe a significant association with rs4970944.

Single nuclei RNA sequencing analysis suggested that *CTSS* expression is enriched in a gene cluster expressing *CD74, DOCK8, C10orf11, ST6GAL1,* and *ARHGAP24*, most of which were reported to be expressed in microglia experimentally (Additional file 1: Fig. S10). We also used a UMAP to visualize the gene expression of *CTSS* and selected genes, including ALS genes (*C9orf72*, *SOD1*), microglial genes (*TREM2*, *CD33*) and the antigen-presenting gene (*HLA-DRB1*). It showed that microglial/antigen-presenting genes (*TREM2, CD33, HLA-DRB1*) and *CTSS* are enriched in the same cluster (Additional file 1: Fig. S11).

## Discussion

Allele-specific methylation (eg, at CpG-SNPs) could help annotate the functional effects of non-coding variants and prioritize candidates as disease risk variants [15]. Hence, we searched for CpG-SNPs associated with ALS age of onset using an innovative strategy combining DNAm and genetic data [34], which could reveal even modest associations often overlooked in genome-wide association studies due to excessive correction for multiple testing leading to the loss of true positive signals. In our large ALS cohort (n = 4629), we detected a modest but significant effect on age of onset of SNPs at an LD-block tagged by rs4970944. The median onset in AA-carriers of rs4970944 was 2 years later than GG-carriers (59 vs 57 years). This association has even a stronger effect size (B = 1.6) in 333 *C9orf72*-carriers, indicating that a larger dataset is needed to search for phenotype modifiers in heterogeneous sporadic patients compared to carriers of the same mutation.

The GTEx database can suggest the link(s) between genetic variations and gene expression levels across a diverse set of human tissues, providing a powerful approach for analyzing eQTLs and inferring the downstream effects of phenotype associated variants [9]. Using the GTEx database, the current study revealed that rs4970944 and its tagging SNPs are eQTLs. In contrast to the protective A-allele, the G-allele of rs4970944 (associated with an earlier ALS onset) is linked to higher *CTSS* expression in cerebellum, which is a critical region in the distributed neural circuits subserving motor control and cognitive processing [28]. Notably, brain expression of *C9orf72* is greatest in cerebellum [25], and *C9orf72*-carriers show a high burden of dipeptide repeat inclusions in cerebellum [21], suggesting that it is an important brain region linking *C9orf72* pathology and the *CTSS*-associated adaptive immune response. However, the connection between rs4970944 and gene expression in other disease-relevant tissues remains to be comprehensively explored. Notably, elevated *CTSS* expression was reported in the anterior lumbar spinal cord of ALS patients [4] and brain tissue of patients with Alzheimer’s Disease [24].

*CTSS* encodes Cathepsin S protein, which is a cysteine endoprotease removing the invariant chain from major histocompatibility complex class II molecules, regulating antigen presentation and immunity [26]. Our single nuclei RNA sequencing analysis and a previous report [29] support the notion that *CTSS* might be a microglia specific gene. Of note, higher expression of another microglia-expressing antigen-presenting gene (*HLA-DRB1*) was also linked to an earlier onset in *C9orf72* patients [34]. Together, it suggests the important role of immune system genes in ALS and other neurodegenerative diseases. For example, several genes causing ALS and/or frontotemporal dementia (*C9orf72, VCP, SQSTM1, OPTN, UBQLN2, GRN, CHMP2B*) affect the autophagic machinery [16]; and some Alzheimer’s Disease genes (*APOE, TREM2, CD33, ABCA7*) are preferentially or exclusively expressed in microglia [17]. Hence, more studies are needed to understand the role of both the innate and adaptive immune pathways in neurodegenerative diseases.

Ongoing international MinE projects (https://www.projectmine.com) aim to characterize both genome and methylome data of ALS cases, which might be used to validate our findings in different ethnic groups. Moreover, it remains to be investigated if the rs4970944-locus plays a role in ALS-related diseases, such as frontotemporal dementia. It would require a large dataset, since the age at onset of frontotemporal dementia is obtained from unaffected family members, in contrast to the more accurate self-reported onset in ALS.

## Conclusions

We identified a 16 Kb LD-block tagged by rs4970944 as a modifier of ALS age of onset. Genotypes of rs4970944 are associated with the DNAm level at the corresponding CpG and linked to cerebellar expression of *CTSS*, highlighting the role of antigen presenting processes in modifying ALS onset. Our findings contribute to understanding the functional consequence of non-coding variants and suggest antigen-presenting processes (eg, involving *CTSS*) as potential drug targets to delay ALS onset. Since inflammation in ALS is a complex phenomenon, future studies may investigate eQTL or splicing QTL data to clarify if other genetic variants may modify disease phenotypes. Independent replication studies are also encouraged to clarify the link between ALS age of onset and the rs4970944-locus.

## Supplementary Information

**Additional file 1:Table S1.** Candidate CpG-SNPs with significant association between their DNAm level and age of onset in 249 ALS patients. **Table S2**. Results of the subgroup analysis in Canadian ALS patients (n=469) and US ALS patients (n=4160). **Table S3**. Bioinformatic annotation of SNPs in strong LD with rs4970944 (R^2^>0.9). **Table S4**. eQTL analysis using the GTEx database revealed significant changes in gene expression associated with rs4970944 in different tissues (normalized effect size (NES) are listed). **Fig. S1**. QQ plot of the genome-wide DNAm study. **Fig. S2**. The 16 Kb LD-block tagged by rs4970944 (chr1:151150857–151166896), including 4 SNPs (rs11204785, rs11807075, rs11299974, rs10888406) in strong LD with rs4970944 (R^2^>0.9). **Fig. S3**. Rs10888406 genotypes are significantly associated with age of onset in ALS patients in the discovery, replication and pooled sample set. Fig. S4. Subgroup analysis in Canadian ALS patients stratified for site of onset, sex and familial history. **Fig. S5**. Subgroup analysis in US ALS patients stratified for site of onset, sex and familial history. **Fig. S6**. Subgroup analysis in US ALS patients stratified for *C9orf72* status. **Fig. S7**. Pooled analysis of the association between rs4970944 genotypes and ALS age of onset. **Fig. S8**. Meta-analysis of the adjusted regression coefficient from the discovery cohort (n=469) and the replication cohort (n=3697) in *C9orf72* negative ALS patients. **Fig. S9**. Rs4970944 genotypes are significantly associated with *CTSS* expression in cerebellum in the GTEx database. **Fig. S10**. The dimension reduction figure (UMAP) of human entorhinal cortex samples. **Fig. S11**. Visualization of *CTSS* and selected genes in the dimension reduction figure (UMAP). Supplementary acknowledgements. Acknowledgements for using the dbGap dataset.

## Data Availability

The datasets generated and/or analysed during the current study are available from the corresponding authors on request.

## Abbreviations

ALS: Amyotrophic lateral sclerosis
SNPs: Single nucleotide polymorphisms
DNAm: DNA methylation
MAF: Minor allele frequency
eQTL: Expression quantitative trait loci
LD: Linkage disequilibrium
NES: Normalized effect size
PCs: Principal components
GTEx: Genotype-tissue expression project
UMAP: Uniform manifold approximation and projection
IQR: Interquartile range
B: Linear regression coefficient Beta
SE: Standard error
