# Supplementary material for: Combined epigenetic/genetic study identified an ALS age of onset modifier
Source: Acta Neuropathol Commun. 2021 Apr 23;9:75. doi: 10.1186/s40478-021-01183-w (PMC8066440; doi:10.1186/s40478-021-01183-w)
Supplement: Supplementary file 1 — Additional file 1:Table S1. Candidate CpG-SNPs with significant association between their DNAm level and age of onset in 249 ALS patients. Table S2. Results of the subgroup analysis in Canadian ALS patients (n=469) and US ALS patients (n=4160). Table S3. Bioinformatic annotation of SNPs in strong LD with rs4970944 (R2>0.9). Table S4. eQTL analysis using the GTEx database revealed significant changes in gene expression associated with rs4970944 in different tissues (normalized effect size (NES) are listed). Fig. S1. QQ plot of the genome-wide DNAm study. Fig. S2. The 16 Kb LD-block tagged by rs4970944 (chr1:151150857–151166896), including 4 SNPs (rs11204785, rs11807075, rs11299974, rs10888406) in strong LD with rs4970944 (R2>0.9). Fig. S3. Rs10888406 genotypes are significantly associated with age of onset in ALS patients in the discovery, replication and pooled sample set. Fig. S4. Subgroup analysis in Canadian ALS patients stratified for site of onset, sex and familial history. Fig. S5. Subgroup analysis in US ALS patients stratified for site of onset, sex and familial history. Fig. S6. Subgroup analysis in US ALS patients stratified for C9orf72 status. Fig. S7. Pooled analysis of the association between rs4970944 genotypes and ALS age of onset. Fig. S8. Meta-analysis of the adjusted regression coefficient from the discovery cohort (n=469) and the replication cohort (n=3697) in C9orf72 negative ALS patients. Fig. S9. Rs4970944 genotypes are significantly associated with CTSS expression in cerebellum in the GTEx database. Fig. S10. The dimension reduction figure (UMAP) of human entorhinal cortex samples. Fig. S11. Visualization of CTSS and selected genes in the dimension reduction figure (UMAP). Supplementary acknowledgements. Acknowledgements for using the dbGap dataset. [file 40478_2021_1183_MOESM1_ESM.pdf]

Electronic supplementary materials

Combined epigenetic/genetic study identified an ALS age of onset modifier

Ming Zhang, PhD[+1-4], Zhengrui Xi, PhD[2], Sara Saez-Atienzar, PhD[5], Ruth Chia, PhD[5], Danielle Moreno, BSc[2], Christine Sato, MSc[2], Mahdi Montazer Haghighi, PhD[2], Bryan J. Traynor, MD, PhD[5], Lorne Zinman, MD[6, 7], and Ekaterina Rogaeva, PhD[+2,7]

[1] Shanghai First Rehabilitation Hospital, School of Medicine, Tongji University, 200090, Shanghai, China

[2] Tanz Centre for Research in Neurodegenerative Diseases, University of Toronto, 60 Leonard Ave., Toronto, ON, Canada, M5T 2S8

[3] Clinical Center for Brain and Spinal Cord Research, Tongji University, 200092, Shanghai, China

[4] Institute for Advanced Study, Tongji University, Shanghai, China

[5] Neuromuscular Diseases Research Section, Laboratory of Neurogenetics, National Institute on Aging, National Institutes of Health, Bethesda, MD 20892, USA

[6] Sunnybrook Health Sciences Centre, 2075 Bayview Ave., Toronto, ON, Canada, M4N 3M5

[7] Division of Neurology, Department of Medicine, University of Toronto, Toronto, Canada

+ Correspondence should be addressed to:

Ekaterina Rogaeva (ekaterina.rogaeva@utoronto.ca)

Tanz Centre for Neurodegenerative Diseases, 60 Leonard Avenue, Toronto, ON, Canada, M5T 2S8; Tel: (416)507-6872; Fax: (416) 603-6435

or

Ming Zhang (mingzhang@tongji.edu.cn)

Shanghai First Rehabilitation Hospital, School of Medicine, Tongji University, Shanghai, China, 200090

Tel: +86 13918066420

**Table of contents**	**Content**	**Page**
Table S1	Candidate CpG-SNPs with significant association between their DNAm level and age of onset in 249 ALS patients.	3
Table S2	Results of the subgroup analysis in Canadian ALS patients (n=469) and US ALS patients (n=4160).	3
Table S3	Bioinformatic annotation of SNPs in strong LD with rs4970944 (R^2>0.9).	3
Table S4	eQTL analysis using the GTEx database revealed significant changes in gene expression associated with rs4970944 in different tissues (normalized effect size (NES) are listed).	4-5
Fig. S1	QQ plot of the genome-wide DNAm study.	5
Fig. S2	The 16 Kb LD-block tagged by rs4970944 (chr1:151150857–151166896), including 4 SNPs (rs11204785, rs11807075, rs11299974, rs10888406) in strong LD with rs4970944 (R^2>0.9).	5
Fig. S3	Rs10888406 genotypes are significantly associated with age of onset in ALS patients in the discovery, replication and pooled sample set.	6
Fig. S4	Subgroup analysis in Canadian ALS patients stratified for site of onset, sex and familial history.	6
Fig. S5	Subgroup analysis in US ALS patients stratified for site of onset, sex and familial history.	7
Fig. S6	Subgroup analysis in US ALS patients stratified for *C9orf72* status.	7
Fig. S7	Pooled analysis of the association between rs4970944 genotypes and ALS age of onset.	8
Fig. S8	Meta-analysis of the adjusted regression coefficient from the discovery cohort (n=469) and the replication cohort (n=3697) in *C9orf72* negative ALS patients.	8
Fig. S9	Rs4970944 genotypes are significantly associated with *CTSS* expression in cerebellum in the GTEx database.	9
Fig. S10	The dimension reduction figure (UMAP) of human entorhinal cortex samples.	9
Fig. S11	Visualization of *CTSS* and selected genes in the dimension reduction figure (UMAP).	10
Supplementary acknowledgements	Acknowledgements for using the dbGap dataset.	10 -11

Table S1. Candidate CpG-SNPs with significant association (in bold) between their DNAm levels and ALS age of onset (n=249). Three CpGs (cg03333305, cg15625495, cg26966808) remained significant after adjustment. Non-Finnish European minor allele frequencies (MAF) were extracted from the gnomAD database.

CpG	p-value	q-value	#Adjusted p-value	*Adjusted q-value	Chr	Position	SNP ID	MAF
**cg03333305**	3.3E-05	0.005	0.0001	**0.04**	12	98812863	rs17227563	0.10
**cg10750264**	0.00011	0.013	0.022	0.49	2	18063316	rs56171351	0.05
**cg07700317**	0.00021	0.021	0.0037	0.25	5	153281406	rs2560030	0.33
**cg20960322**	0.00036	0.028	0.0034	0.24	1	182002021	rs7542928	0.16
**cg15625495**	0.00044	0.033	0.0002	**0.04**	1	151163317	rs4970944	0.32
**cg04610028**	0.00057	0.037	0.023	0.50	19	8464538	rs2967607	0.27
**cg15959464**	0.00060	0.039	0.037	0.58	21	46763239	rs73238114	0.2
**cg13585749**	0.00064	0.040	0.068	0.65	2	875438	rs4075904	0.16
**cg10584449**	0.00079	0.047	0.003	0.24	8	133984058	rs2076740	0.35
**cg26966808**	0.00003	0.004	0.0002	**0.048**	17	4502245	rs4790206	0.34

Adjusted for sex, site of onset, DNAm-age acceleration and 5 Principal Components (PC1-PC5)
* q-values (at a false discovery rate <0.05) were estimated based on p-values adjusted for sex, site of onset, DNAm-age acceleration and PC1-PC5

Table S2. Results of the subgroup analysis in Canadian ALS patients (n=469) and US ALS patients (n=4160). Patients were stratified by sex, site of onset or family history. The MWU test was used to analyze the age of onset difference in each subgroup.

**Subgroups**	**Canadian ALS patients**		**US ALS patients**	
	**Age of onset (mean, years)**	**P-value**	**Age of onset (mean, years)**	**P-value**
**Sex**		0.7		3.1×10^{-13}
Male	60.3		59	
Female	59.9		56.2	
**Site of onset**		0.0016		2.2×10^{-16}
Limb	59		55.8	
Bulbar	63.1		60.8	
**Family history**		0.0005		0.001
Familial ALS	54.6		55.4	
Sporadic ALS	61.1		57.6	

Table S3. Bioinformatic annotation of SNPs in strong linkage disequilibrium with rs4970944 (R^2>0.9). TFBS represents transcriptional factor binding site and MAF is minor allele frequency.

Rs-number	Coordinates	Alleles	MAF	Distance (bp)	Dprime	R^2	TFBS	DNase I hypersensitivity sites
**rs11204785**	chr1:151150857	T/C	0.3221	-12460	1	0.96	no	no
**rs11807075**	chr1:151153806	C/T	0.3221	-9511	1	0.96	no	no
**rs4970944**	chr1:151163317	G/A	0.3131	0	1	1	no	no
**rs11299974**	chr1:151163481	G/-	0.3141	164	1	0.99	no	no
**rs10888406**	chr1:151166896	G/T	0.3131	3579	1	1	no	no

Table S4. eQTL analysis using the GTEx database revealed significant changes in gene expression associated with rs4970944 in different tissues (normalized effect size (NES) are listed).

**Gencode Id**	**Gene**	**Location (hg19)**	**P-Value**	**NES**	**Tissue**
ENSG00000163131.10	*CTSS*	chr1:150702664 -150738254	0.00018	-0.31	Cerebellum
ENSG00000143452.15	*HORMAD1*	chr1:150670536 -150693371	8.40E-07	-0.30	Skin - Sun Exposed (Lower leg)
ENSG00000143452.15	*HORMAD1*	chr1:150670536 -150693371	0.000022	-0.29	Nerve - Tibial
ENSG00000143452.15	*HORMAD1*	chr1:150670536 -150693371	0.000004	-0.28	Adipose - Subcutaneous
ENSG00000143452.15	*HORMAD1*	chr1:150670536 -150693371	0.00003	-0.28	Cells - Cultured fibroblasts
ENSG00000143452.15	*HORMAD1*	chr1:150670536 -150693371	0.00009	-0.28	Esophagus - Mucosa
ENSG00000143457.10	*GOLPH3L*	chr1:150618706 -150669630	0.000002	-0.24	Skin - Sun Exposed (Lower leg)
ENSG00000143452.15	*HORMAD1*	chr1:150670536 -150693371	0.0002	-0.24	Skin - Not Sun Exposed (Suprapubic)
ENSG00000163131.10	*CTSS*	chr1:150702664 -150738254	3.10E-08	-0.18	Thyroid
ENSG00000261168.1	*RP11-68I18.10*	chr1:151102551 -151104086	0.00004	-0.15	Testis
ENSG00000163131.10	*CTSS*	chr1:150702664 -150738254	0.00004	-0.14	Heart - Atrial Appendage
ENSG00000143387.12	*CTSK*	chr1:150768684 -150780736	0.00002	-0.13	Whole Blood
ENSG00000163131.10	*CTSS*	chr1:150702664 -150738254	0.00002	-0.13	Muscle - Skeletal
ENSG00000163131.10	*CTSS*	chr1:150702664 -150738254	0.000005	-0.11	Artery - Tibial
ENSG00000163131.10	*CTSS*	chr1:150702664 -150738254	0.0004	-0.11	Nerve - Tibial
ENSG00000143437.20	*ARNT*	chr1:150782189 -150849075	0.00008	-0.10	Artery - Tibial
ENSG00000143373.17	*ZNF687*	chr1:151253998 -151264652	0.0004	-0.09	Nerve - Tibial
ENSG00000159352.15	*PSMD4*	chr1:151227210 -151239936	0.00003	0.06	Whole Blood
ENSG00000159352.15	*PSMD4*	chr1:151227210 -151239936	0.00005	0.08	Nerve - Tibial
ENSG00000159352.15	*PSMD4*	chr1:151227210 -151239936	2.80E-08	0.09	Artery - Tibial
ENSG00000159352.15	*PSMD4*	chr1:151227210 -151239936	0.000003	0.09	Thyroid
ENSG00000159352.15	*PSMD4*	chr1:151227210 -151239936	0.00003	0.10	Esophagus - Gastroesophageal Junction
ENSG00000143398.19	*PIP5K1A*	chr1:151171028 -151222007	0.0004	0.10	Skin - Not Sun Exposed (Suprapubic)
ENSG00000143398.19	*PIP5K1A*	chr1:151171028 -151222007	0.00002	0.12	Skin - Sun Exposed (Lower leg)
ENSG00000143398.19	*PIP5K1A*	chr1:151171028 -151222007	0.0002	0.13	Artery - Aorta
ENSG00000143443.9	*C1orf56*	chr1:151020227 -151023896	0.0003	0.13	Skin - Sun Exposed (Lower leg)
ENSG00000143398.19	*PIP5K1A*	chr1:151171028 -151222007	9.40E-08	0.16	Artery - Tibial

ENSG00000143398.19	*PIP5K1A*	chr1:151171028 -151222007	6.70E-11	0.18	Nerve - Tibial
ENSG00000159352.15	*PSMD4*	chr1:151227210 -151239936	0.000005	0.19	Putamen (basal ganglia)

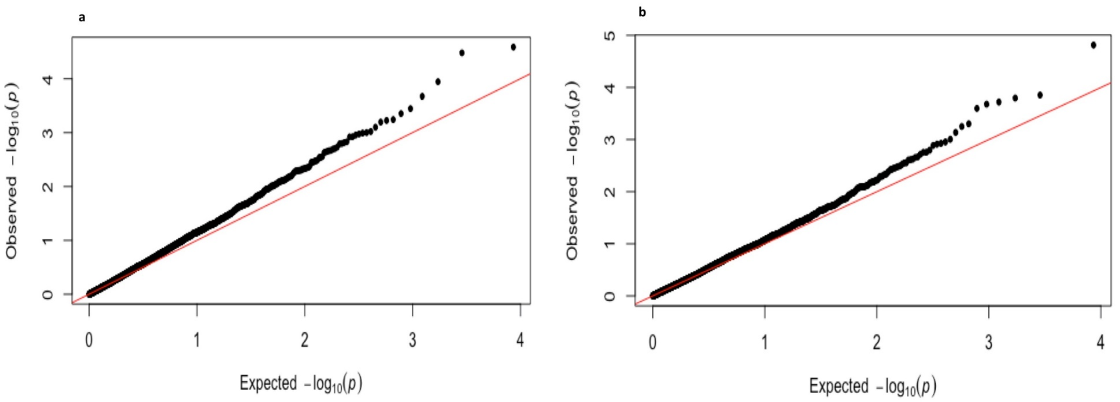

Fig. S1. QQ plot of the genome-wide DNAm study in 249 ALS patients for **(a)** unadjusted p-values and **(b)** p-values adjusted for gender, site of onset, DNAm-age acceleration and the top 5 PCs. The genomic inflation factor (lambda) is 1.16 for unadjusted p-values and 1.06 for adjusted p-values.

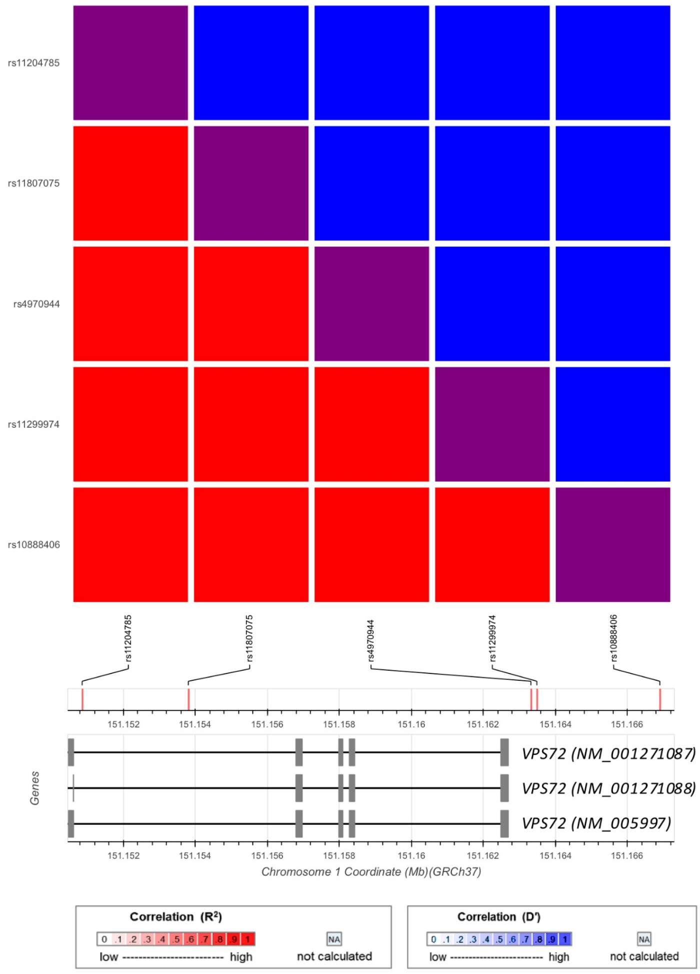

Fig. S2. The 16 Kb LD-block tagged by rs4970944 (chr1:151150857–151166896), including 4 SNPs (rs11204785, rs11807075, rs11299974, rs10888406) in strong LD with rs4970944 (R^2>0.9). It partially overlaps the *VPS72* gene.

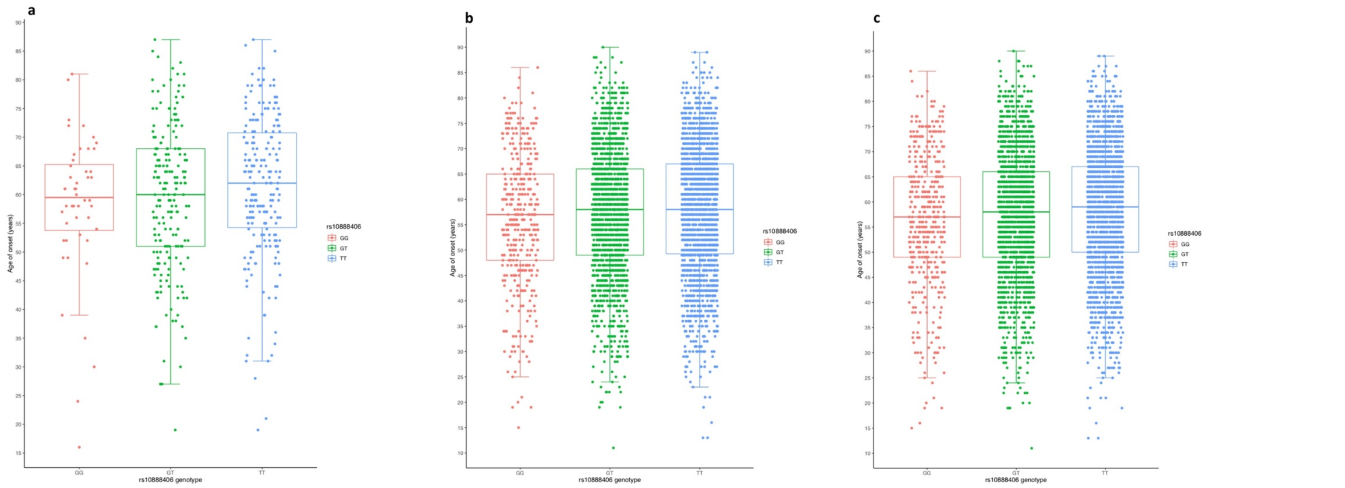

Fig. S3. Rs10888406 genotypes are significantly associated with age of onset in ALS patients in the **(a)** discovery cohort (adjusted P=0.026), **(b)** replication cohort (adjusted P=0.007) and **(c)** pooled sample set (n=4629, P=0.001, adjusted for sex, site of onset and family history).

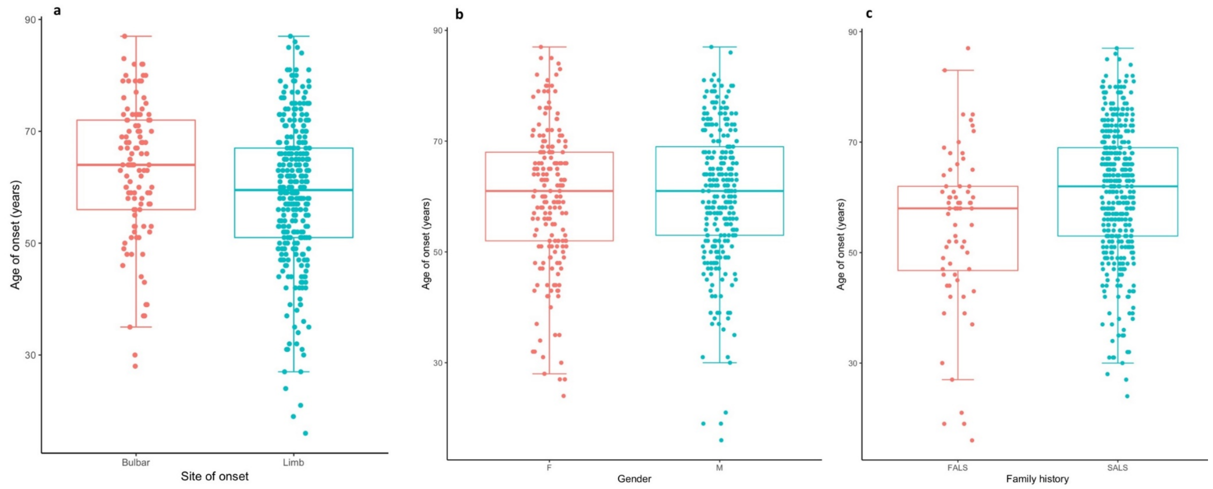

Fig. S4. Subgroup analysis in Canadian ALS patients stratified for (a) site of onset, (b) sex and (c) familial history. Age of onset is significantly different between patients with bulbar and limb onset (P=0.0016, MWU test), familial and sporadic ALS patients (P=0.0005, MWU test). There is no significant difference in age of onset between male and female patients (P=0.7, MWU test).

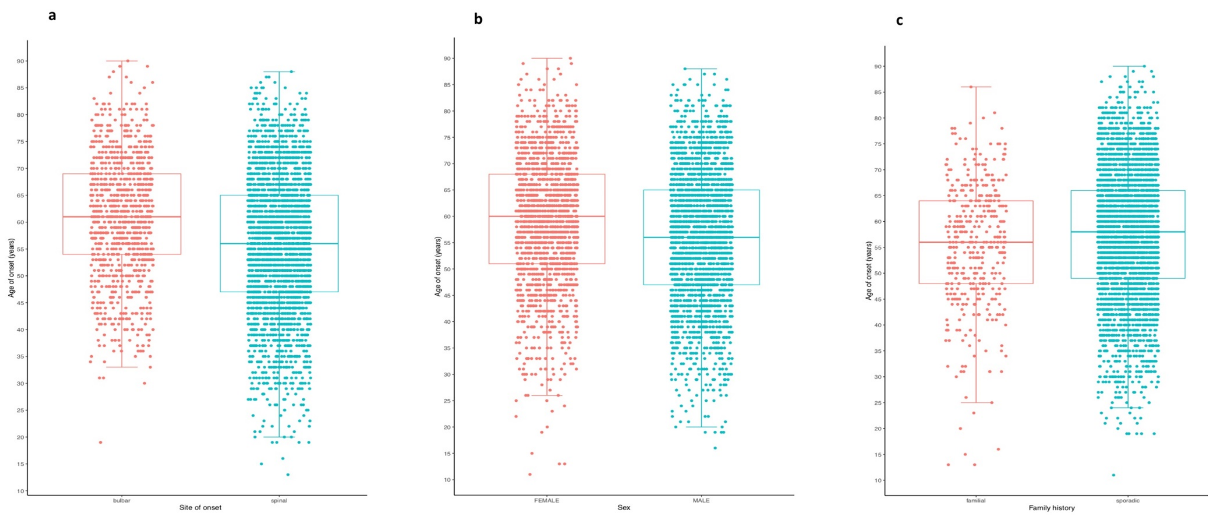

Fig. S5. Subgroup analysis in US ALS patients stratified for (a) site of onset, (b) sex and (c) familial history. Patients with bulbar onset had significantly later onset than patients with limb onset ($P=2.2\times 10^{-16}$). Familial ALS patients had a significantly earlier onset than sporadic ALS patients (P=0.001). Female ALS patients had significantly later onset than male patients ($P=3.1\times10^{-13}$).

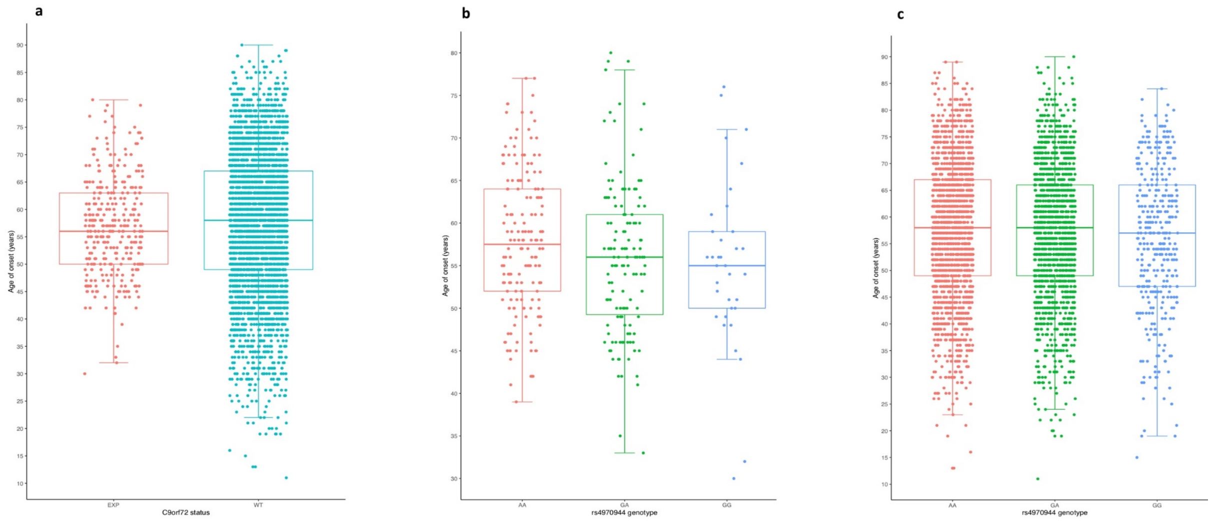

Fig. S6. Subgroup analysis in US ALS patients stratified for *C9orf72* status. (a) There is no significant difference in age of onset between *C9orf72* carriers (EXP) and *C9orf72* negative patients (WT) (P=0.08, MWU test, n=333 vs 3697). Genotypes of rs4070944 are significantly associated with age of onset in both **(b)** *C9orf72* carriers (adjusted P=0.025, B=1.6, SE=0.7) and **(c)** *C9orf72* negative patients (adjusted P=0.015, B=0.78, SE=0.3).

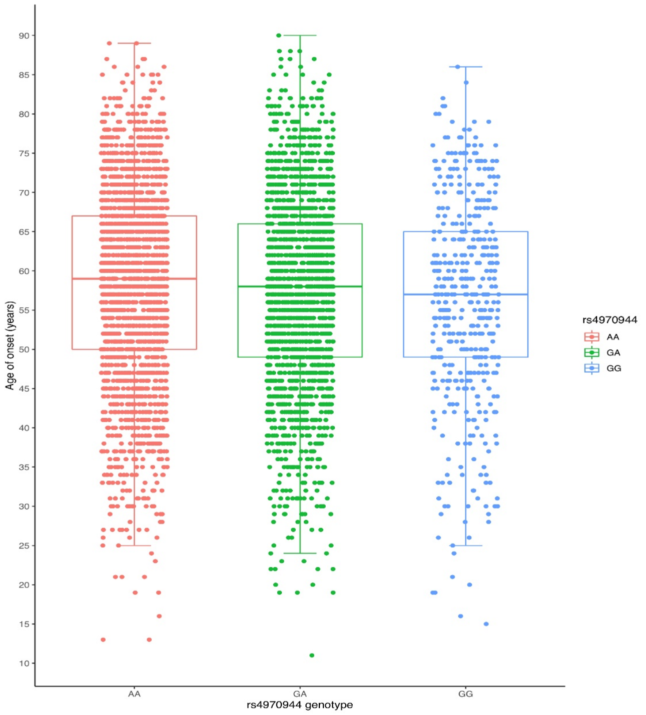

Fig. S7. Pooled analysis of the association between rs4970944 genotypes and ALS age of onset. In the pooled ALS sample set (n=4629), rs4970944 genotypes are significantly associated with age of onset (P=0.001, adjusted for sex, site of onset and family history). Each A-allele is associated with a 0.9 year later onset.

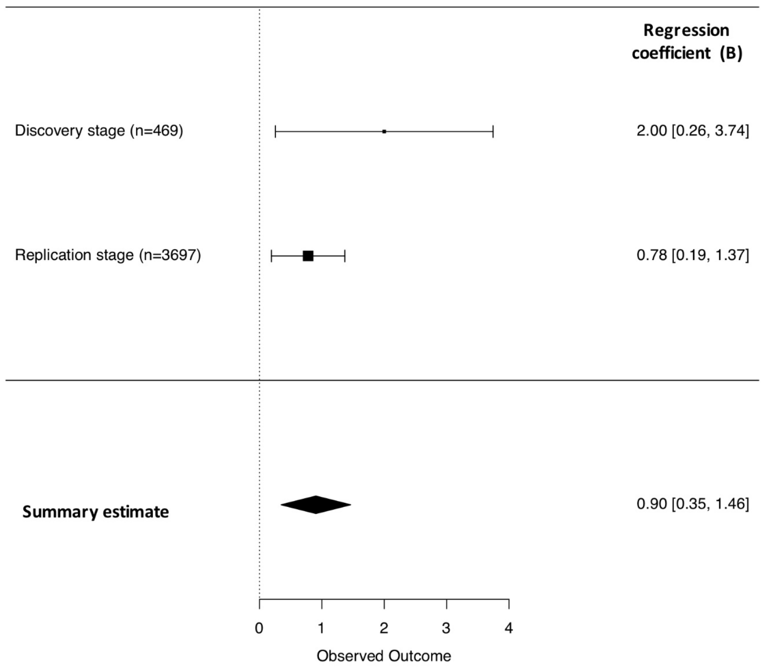

Fig. S8. Meta-analysis of the adjusted regression coefficient from the discovery cohort (n=469) and the replication cohort (n=3697) in *C9orf72* negative ALS patients. It confirmed the significant association between rs4970944 and age of onset (pooled B=0.9, P=0.0015) in *C9orf72* negative ALS patients.

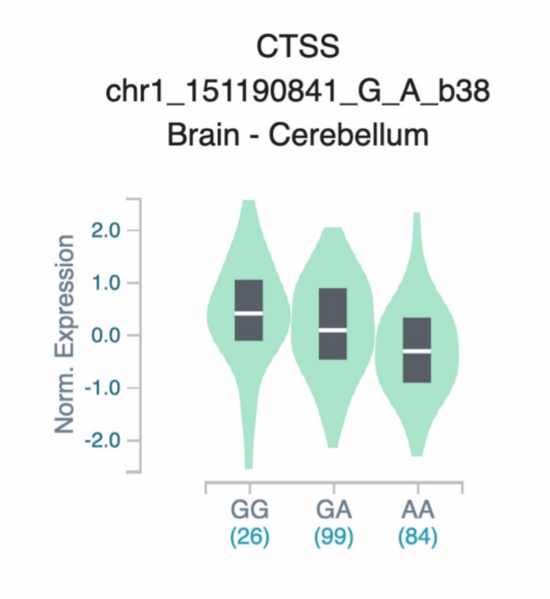

Fig. S9. Rs4970944 genotypes are significantly associated with *CTSS* expression in cerebellum in GTEx database (P=0.00018, NES=-0.31). It suggests that the A-allele of rs4970944 is associated with a lower expression of *CTSS*.

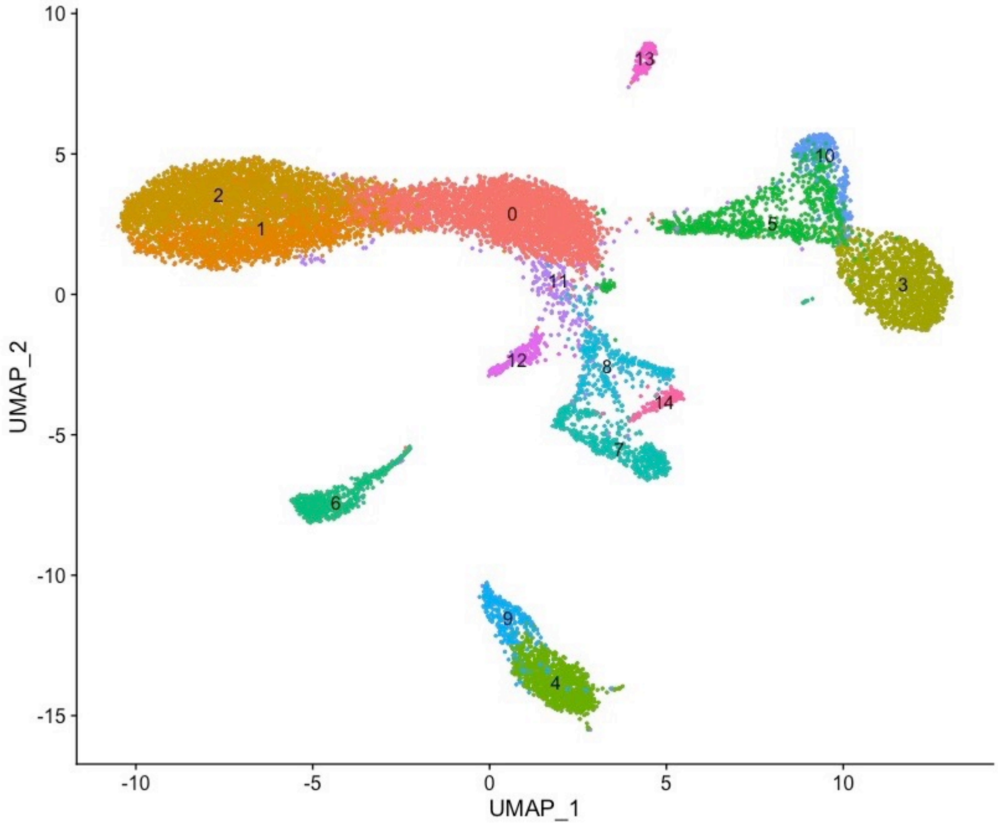

Fig. S10. The dimension reduction figure (UMAP) of human entorhinal cortex samples (n=8) using a publicly available dataset (GEO: GSE138852). 15 clusters are presented. *CTSS* expression is enriched in cluster 6 specifically expressing *CD74/DOCK8/C10orf11/ST6GAL1/ARHGAP24*; all of these genes except for *C10orf11* were reported to be expressed in microglia experimentally.

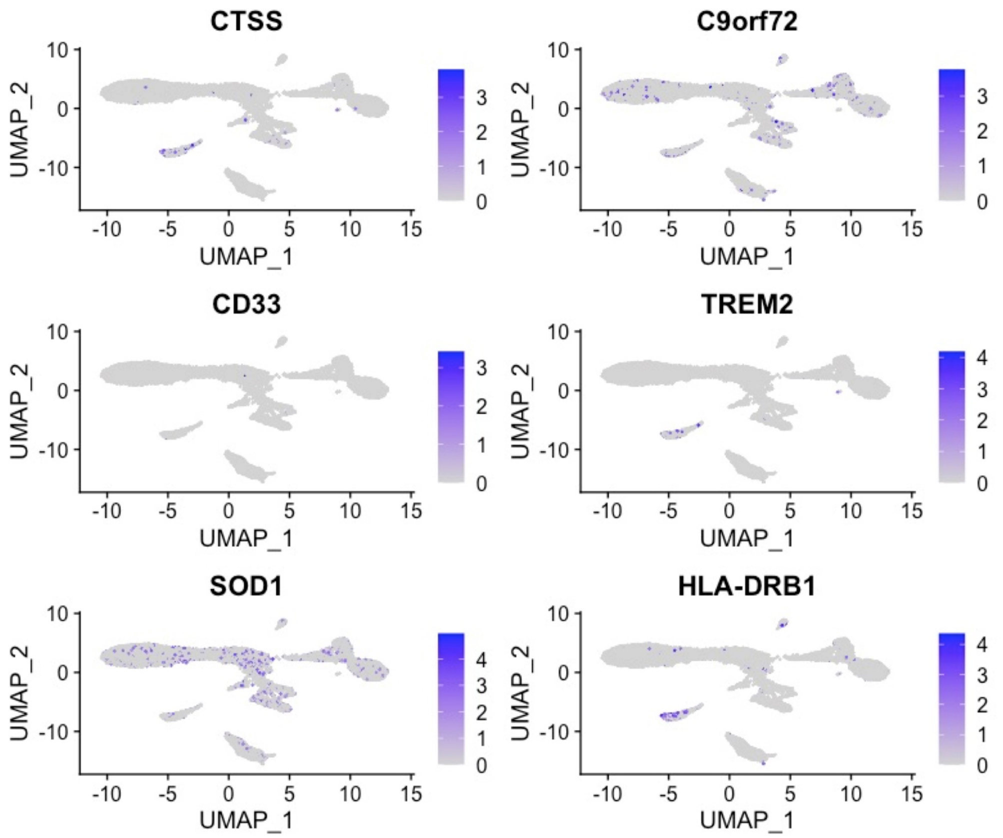

Fig. S11. Visualization of *CTSS* expression and selected genes (ALS genes*: C9orf72* and *SOD1*, microglial genes: *TREM2* and *CD33*, the antigen presenting gene: *HLA-DRB1*) in the dimension reduction figure (UMAP).

Supplementary acknowledgements

The dataset(s) used for the analyses described in this manuscript were obtained from the Age-Related Eye Disease Study (AREDS) Database found at https://www.nei.nih.gov/research/clinical-trials/age-related-eye-disease-study-areds through dbGaP accession number phs000001.v3.p1. Funding support for AREDS was provided by the National Eye Institute (N01-EY-0-2127). We would like to thank the AREDS participants and the AREDS Research Group for their valuable contribution to this research.

The Framingham Heart Study is conducted and supported by the National Heart, Lung, and Blood Institute (NHLBI) in collaboration with Boston University (Contract No. N01-HC-25195 and HHSN268201500001I). This manuscript was not prepared in collaboration with investigators of the Framingham Heart Study and does not necessarily reflect the opinions or views of the Framingham Heart Study, Boston University, or NHLBI. Funding to support the Omni cohort recruitment, retention and examination was provided by NHLBI Contract N01-HC-25195 and HHSN268201500001I, as well as NHLBI grants R01-HL070100, R01-HL076784, R01-HL-49869, and U01-HL-053941.

Research support to collect data and develop an application to support this project was provided by 3P50CA093459, 5P50CA097007, 5R01ES011740, and 5R01CA133996.

The WHI program is funded by the National Heart, Lung, and Blood Institute, National Institutes of Health, U.S. Department of Health and Human Services through contracts HHSN268201600018C, HHSN268201600001C, HHSN268201600002C, HHSN268201600003C, and HHSN268201600004C. This manuscript was not prepared in collaboration with investigators of the WHI, has not been reviewed and/or approved by the Women's Health Initiative (WHI), and does not necessarily reflect the opinions of the WHI investigators or the NHLBI. Funding support for WHI GARNET was provided through the NHGRI Genomics and Randomized Trials Network (GARNET) (Grant Number U01 HG005152). Assistance with phenotype harmonization and genotype cleaning, as well as with general study coordination, was provided by the GARNET Coordinating Center (U01 HG005157). Assistance with data cleaning was provided by the National Center for Biotechnology Information. Funding support for genotyping, which was performed at the Broad Institute of MIT and Harvard, was provided by the NIH Genes, Environment and Health Initiative [GEI] (U01 HG004424). The datasets used for the analyses described in this manuscript were obtained from

dbGaP at http://www.ncbi.nlm.nih.gov/sites/entrez?db=gap through dbGaP accession phs000001, phs000007, phs000187, phs000196, phs000200, phs000292, phs000304, phs000315, phs000368, phs000372, phs000394, phs000397, phs000404, phs000421, phs000428, phs000615, phs000675, phs000801, and phs000869.

Funding support for the Genes and Blood Clotting Study was provided through the NIH/NHLBI (R37 HL039693). The Genes and Blood Clotting Study is one of the Phase 3 studies as part of the Gene Environment Association Studies (GENEVA) under GEI. Assistance with genotype cleaning was provided by the GENEVA Coordinating Center (U01 HG004446). Funding support for DNA extraction and genotyping, which was performed at the Broad Institute, was provided by NIH/NHLBI (R37 HL039693). Additional support was provided by the Howard Hughes Medical Institute.

The dataset(s) used for the analyses described in this manuscript were obtained from the database of Genotype and Phenotype (dbGaP) found at http://www.ncbi.nlm.nih.gov/gap through dbGaP accession number phs000368. Samples and associated phenotype data for the Genome-Wide Association Scan [GWAS] of Polycystic Ovary Syndrome Phenotypes were provided by Andrea Dunaif, M.D.

The authors acknowledge the contribution of data from Genetic Architecture of Smoking and Smoking Cessation accessed through dbGAP. Funding support for genotyping, which was performed at the Center for Inherited Disease Research (CIDR), was provided by 1 X01 HG005274-01. CIDR is fully funded through a federal contract from the National Institutes of Health to The Johns Hopkins University, contract number HHSN268200782096C. Assistance with genotype cleaning, as well as with general study coordination, was provided by the Gene Environment Association Studies (GENEVA) Coordinating Center (U01 HG004446). Funding support for collection of datasets and samples was provided by the Collaborative Genetic Study of Nicotine Dependence (COGEND; P01 CA089392) and the University of Wisconsin Transdisciplinary Tobacco Use Research Center (P50 DA019706, P50 CA084724).

The dataset(s) used for the analyses described in this manuscript were obtained from the Genetics of Fuchs' Endothelial Corneal Dystrophy (FECD) Study through dbGaP accession number phs000421. The grants that have funded the enrollment of the cases and controls to be used in this GWAS are:R01EY016514 (DUEC, PI: Gordon Klintworth), R01EY016482 (CWRU, PI: Sudha Iyengar), and 1X01HG006619-01 (PI: Sudha Iyengar, Natalie Afshari). We would like to thank the FECD participants and the FECD Research Group for their valuable contribution to this research

The authors acknowledge the contribution of data from CIDR-NIDA Study of HIV Host Genetics accessed through dbGAP. Funding support for genotyping, which was performed at the Center for Inherited Disease Research (CIDR), was provided by 1 X01 HG005275-01A1. CIDR is fully funded through a federal contract from the National Institutes of Health to The Johns Hopkins University, contract number HHSN268200782096C. Funding support for collection of datasets and samples was provided by NIDA grants R01DA026141(Johnson); R01DA004212 (Watters); U01DA006908 (Watters); R01DA009532 (Bluthenthal); as well as the San Francisco Department of Public Health; SAMHSA; and HRSA.

The Genome-Wide Association Study (GWAS) of Non-Hodgkin Lymphoma (NHL) project was supported by the intramural program of the Division of Cancer Epidemiology and Genetics (DCEG), National Cancer Institute (NCI), National Institutes of Health (NIH). The datasets have been accessed through the NIH database for Genotypes and Phenotypes (dbGaP) under accession # phs000801. A full list of acknowledgements can be found in the supplementary note (Berndt SI et al., Nature Genet., 2013, PMID: 23770605).

This study made use of data generated by investigators in the BEACON consortium through a grant funded by the US National Institutes of Health (NIH) (RO1CA136725) to Thomas L. Vaughan and David C. Whiteman (multiple PIs). In support of this work, T.L.V. was also supported by NIH grant KO5CA124911 and D.C.W. by a Future Fellowship grant FT0990987 from the Australia Research Council. Additional collaborators, sources of support and origin of the data and biospecimens are listed in the following publication: Levine DM, Ek WE, Zhang R, Liu X, Onstad L, Sather C, et al. A genome-wide association study identifies new susceptibility loci for esophageal adenocarcinoma and Barrett's esophagus. Nat Genet. 2013 Dec;45(12):1487–93.
